# Sharing the filmic experience - The physiology of socio-emotional processes in the cinema

**DOI:** 10.1371/journal.pone.0223259

**Published:** 2019-10-18

**Authors:** Laura Kaltwasser, Nicolas Rost, Martina Ardizzi, Marta Calbi, Luca Settembrino, Joerg Fingerhut, Michael Pauen, Vittorio Gallese

**Affiliations:** 1 Berlin School of Mind and Brain, Humboldt-Universität zu Berlin, Berlin, Germany; 2 Department of Medicine and Surgery, Unit of Neuroscience, University of Parma, Parma, Italy; 3 Institut für Philosophie, Humboldt-Universität zu Berlin, Berlin, Germany; 4 Department of Art History Columbia University, Italian Academy for Advanced Studies, Columbia University, New York, NY, United States of America; University of Bologna, ITALY

## Abstract

As we identify with characters on screen, we simulate their emotions and thoughts. This is accompanied by physiological changes such as galvanic skin response (GSR), an indicator for emotional arousal, and respiratory sinus arrhythmia (RSA), referring to vagal activity. We investigated whether the presence of a cinema audience affects these psychophysiological processes. The study was conducted in a real cinema in Berlin. Participants came twice to watch previously rated emotional film scenes eliciting amusement, anger, tenderness or fear. Once they watched the scenes alone, once in a group. We tested whether the vagal modulation in response to the mere presence of others influences explicit (reported) and implicit markers (RSA, heart rate (HR) and GSR) of emotional processes in function of solitary or collective enjoyment of movie scenes. On the physiological level, we found a mediating effect of vagal flexibility to the mere presence of others. Individuals showing a high baseline difference (alone vs. social) prior to the presentation of film, maintained higher RSA in the alone compared to the social condition. The opposite pattern emerged for low baseline difference individuals. Emotional arousal as reflected in GSR was significantly more pronounced during scenes eliciting anger independent of the social condition. On the behavioural level, we found evidence for emotion-specific effects on reported empathy, emotional intensity and Theory of Mind. Furthermore, people who decrease their RSA in response to others’ company are those who felt themselves more empathically engaged with the characters. Our data speaks in favour of a specific role of vagal regulation in response to the mere presence of others in terms of explicit empathic engagement with characters during shared filmic experience.

## Introduction

The youngest film adaptation of Stephen King’s ‘It’ brought more people into the cinema than any other horror movie in history. Upon release, the film has grossed over $653 million worldwide and set numerous box office records (Calfas, Jennifer. ‴It' Shatters Box Office Records With Massive Opening Weekend". Time. Retrieved 2018-09-11). How can we explain that people prefer to experience the narrative around harrowing clown ‘Pennywise’ in the cinema and not at home? Spectators seem to enjoy many genres such as sports, drama, comedy or pornography alone at home, but they prefer to immerse into horror in societal company. Scientists in the field of aesthetics, cultural and media studies claim that the future of cinema is built upon horror movies as ‘It’, suggesting that intensive societal experience lies in sharing negative emotions such as fear[1–5]. The aim of this study was to reveal the underlying physiological mechanisms of shared experience during film reception in a cinema, focusing on filmic experience as shared activity based on the intention to jointly attend to the same object–the movie[6].

The effect of social conditions on experience of film and filmic narrative is to date an under investigated phenomenon. Given the different viewing formats of today’s films between cinema, home screen, individual screen (Laptop, Ipad), understanding the impact of the social situation on the emotional and cognitive engagement with the character and narrative constitutes a central element in a more fully developed and ecologically valid theory of film engagement. The limited literature on this topic provides contrasting results.

Dunand and colleagues[7] report that social company amplifies aggression expression triggered by a movie. In order to examine this audience effect on participants viewing filmed violence, male participants watched an aggressive or a neutral movie either alone, accompanied by a passive confederate, or by someone reacting to the movie. Beyond the usual instigation effect of filmed violence, the authors observed that participants displayed their aggressive behaviour most when accompanied by an active confederate during the violent movie.

Importantly, such an effect of *social facilitation* seems to depend on the real physiological presence of another, since a mere hypothetical fellow participant in an adjacent room has no effect on emotional processes elicited through movie clips[8].

Egermann and colleagues[9] investigated whether listening to music in a group setting alters the emotion felt by listener. They reported no difference between the retrospective emotion ratings provided by participants listening to a piece of music alone or with others. Interestingly, skin conductance responses were significantly higher during the solitary listening condition, in which there was a non-significant trend towards experiencing more chills. These results led the authors to conclude that music listening was more arousing alone in contrast with the social facilitation theory, which predicts that the mere presence of others leads to increased arousal[10].

Since movies contact us at an embodied level [11], we can study physiological markers of the shared experience during film reception when spectators identify themselves with characters in a movie. As the multisensory experience of film can induce a wide range of affective responses, several studies have used film clips, especially those with an intense emotional content[12–17], to investigate the physiological reactions of the Autonomic Nervous System (ANS) to emotions. Though few published studies have compared film sets with other emotion elicitation techniques, some evidence suggests that film clips are able to evoke emotions–both in forms of subjective and physiological changes–more successfully than other methods[18–20]. This greater capability in inducing physiological reactions is related to several advantages that movies have in comparison to other emotion-inducing methods (i.e., high degree of standardization [19], multimodality[20], greater ecological validity, more contextual information[21, 22] more sustained induced affective state [23]).

The ANS is primarily made up of Sympathetic Nervous System (SNS) and Parasympathetic Nervous System branches (PNS) that together dynamically regulate internal viscera including cardiac, respiratory, and glandular systems. In general, the SNS is a catabolic system associated with physiological activation (i.e., increased arousal or mobilizing responses) and the PNS an anabolic system associated with restoration and repair (i.e., decreased arousal or rest). Both branches work in tandem, and dynamically change as they regulate the body in preparation for and response to current endogenous and exogenous environmental conditions. For these reasons, the ANS is well suited to investigate different physiological response patterns elicited through emotional film. Across the different indexes of ANS, characterized by the well-known directional fractionation property, the most commonly assessed indices of ANS branches activation are based on cardiovascular or electrodermal responses. Cardiovascular measures include heart rate (HR), blood pressure, total peripheral resistance, cardiac output, pre-ejection period, and heart rate variability. Each of these measures varies in terms of whether it primarily reflects sympathetic activity, parasympathetic activity, or both. Electrodermal responding is typically quantified in terms of Galvanic skin response (GSR). In the present study, we measured respiratory sinus arrhythmia (RSA), i.e. periodic fluctuations in heart rate (HR) in phase with breathing, that is closely linked to parasympathetic activity[24, 25], HR and GSR–predominantly reflecting sympathetic activity. RSA reflects high frequency heart rate variability and has been shown to be modulated by social communication such as displaying facial expressions, making eye contact, expressing prosody, self-regulatory behaviour as well as prosocial orientation [26–34].

According to the *polyvagal theory*[28, 29], RSA is generated in functional distinct vagal systems that first evolved in the brainstem of mammals. In more detail, it maintains that there are three main anatomically and physiologically subsystems, whose function is linked to immobilization, mobilization, and social communication, respectively. The last subsystem, unique to mammals, rapidly regulates the cardiac output through the myelinated vagal fibres: if the environment is perceived as safe, it decreases HR to inhibit ineffective defensive behaviours and to recruit the subsystem associated with social communication. The dynamic influence of this “vagal brake” can be monitored by quantifying the amplitude of RSA. Vagally mediated heart rate deceleration, with corresponding increases in RSA, is expected to occur when an organism attends to and engages with an environmental stimulus or when it relaxes in a safe environment. In humans, this may often be driven by self-regulatory efforts designed to facilitate such engagement or relaxation and may be accompanied by corresponding positive mood states[35, 36]. In contrast, decreases in RSA are expected when an organism responds to the environment with a fight-or-flight activation pattern, which involves an increased sympathetic activation[32]. Thus, reductions in RSA are assumed to indicate physiological responses to stressors and to be accompanied by negative mood states due to the presence of those stressors[37–39]. A bulk of studies suggested that tonic RSA, measured during a rest condition, is a valid implicit index of individual differences in social predisposition, emotional expressiveness and self-regulation skills. Specifically, the higher the tonic RSA, the higher the individual social engagement propensity and emotion regulation abilities[40–42]. Furthermore, individual differences in vagal flexibility are useful physiological predictor of social sensitivity. Indeed, individuals with greater vagal flexibility respond to dynamic social feedback in a more context-sensitive manner than do individuals with less vagal flexibility[43]. Proceeding from this evidence, the vagal flexibility in response to others’ mere presence in a rest condition could play a crucial role in determining how social company affects individual responses, both at an explicit and physiological level, to solitary or collective enjoyment of film clips.

GSR is measured through the resistance to weak current (.5 V) applied to the skin. Afferent neurons from the sympathetic axis of the autonomic nervous system innervate eccrine sweat (sudomotor) glands, and their activity modulates conductance of an applied current. Instantaneous sympathetic arousal, transmitted via individual firing bursts of the sudomotor nerve, provokes an increase in the amplitude of GSR[44, 45]. GSR is a widely used and sensitive index of emotion-related autonomic arousal[46–48].

In the present study, we measured HR, RSA and GSR in healthy participants while they were watching 2-minute long film clips either alone (alone condition) or with three unfamiliar confederates in a gender-balanced group (social condition). The film clips were selected in order to have eight clips eliciting different emotions (anger, fear, amusement, tenderness) and two emotionally neutral ones, providing an experimental design which is balanced according to valence. Before the beginning of the two conditions, a rest period was recorded in order to measure tonic RSA flexibility between solitary or collective contexts. After the presentation of each clip, the participants answered questions related to empathy, emotional intensity, Theory of Mind (ToM), and memory, as well as about the quality and intensity of emotions felt during the stimulus presentation.

Based on the literature reviewed above, regarding the physiological reactivity to the mere presence of others we predicted three possible scenarios. People could increase their tonic RSA in the social condition with respect to the alone condition, showing an augmented social disposition in response to the mere presence of unfamiliar others. Vice versa, people could decrease their tonic RSA in the social condition with respect to the alone condition, showing that this social context is perceived as stressful. Lastly, also an absence of modulation between the two conditions could be hypothesized suggesting that people were not sensitive to the social presence of others. If the vagal flexibility in response to others’ mere presence in the rest condition influences how social company affects the vagal modulation during the enjoyment of film clips, we expected significant differences between social and alone conditions in the three groups. Specifically, we hypothesized that people who feel comfortable in response to the mere presence of others may show a further increase in their RSA modulation, as well as a decrease in GSR, during the collective enjoyment of film clips. Differently, people who feel the presence of others as a stressor may reduce their RSA modulation and increase their GSR during the filmic shared experience. We did not exclude the possibility of an interaction between individual vagal modulation to the mere presence of others and the emotional content of the scenes enjoyed in solitary or collective context. For example, people who increase their vagal modulation in response to the mere presence of others may also show a higher RSA accompanied by a lower GSR during the collective enjoyment of negative film clips than the solitary experience of the same film clips. This result could partially explain why some people prefer to immerse into horror in societal company. Conversely, people who decrease their vagal modulation in response to the mere presence of others may also show a higher RSA accompanied by a lower GSR during the collective enjoyment of positive and affiliative film clips (e.g., amusing movies) than the solitary experience of the same.

Considering the explicit evaluation of the different film clips, an increased level of empathy and felt emotional intensity in the social condition, due to the presence of emotional contagion, was expected especially among people with high social disposition to the mere presence of others. Differently, regardless the individual social disposition at rest, better performance in ToM and memory tasks were anticipated in the alone condition.

## Method

### Participants

The audience consisted of *N* = 39 participants (20 female) aged between 20 and 36 years (*M* = 27.3; *SD* = 4.4). The total sample size exceeded the minimum amount required (*N* = 27) estimated by means of statistical a priori sample size calculation, obtained for repeated-measures ANOVA considering both within and between interactions (1-ß = 0.95, α = 0.05 and effect size f = 0.25). Post-hoc power estimation analysis conducted for repeated-measures ANOVA considering both within and between interactions including the actual effect size of our main interaction condition*baseline RSA (f = 0.88) and the final sample size split in three groups (n. = 39) confirmed the high achieved statistical power (1-ß = 0.99). Participants did not have a history of psychiatric, neurological nor cardiac disease, neither were they heavy smokers or abusive of drugs or alcohol. They were not on any medication (except oral contraceptives) and asked to refrain from caffeine on the day of testing. All participants were recruited through several English speaking teaching institutions as well as community websites and had either native or very good English skills (minimum level C2). The study was approved by the Ethics committee of the Psychology Institute of Humboldt-Universität zu Berlin and it was in line with the Declaration of Helsinki 2013. Informed consent was obtained prior to examination. Participants received 24 € compensation for a total testing of maximally three hours within two sessions (two weeks apart).

### Procedure

**Fig 1:**
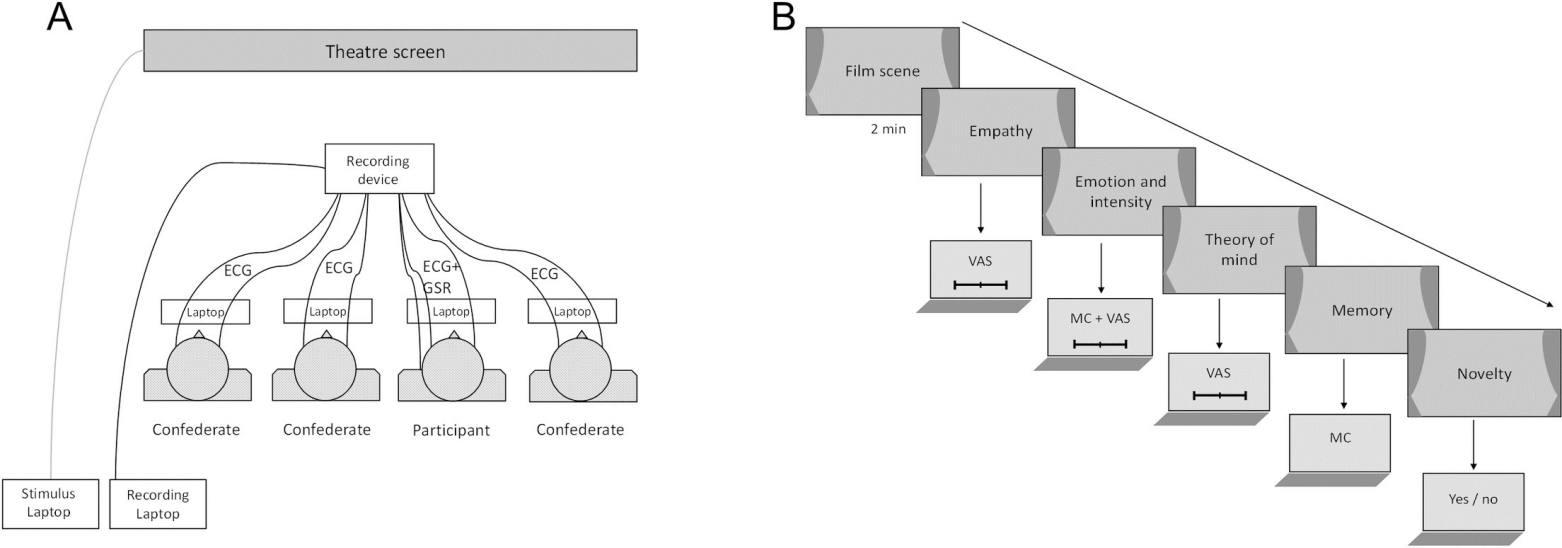
Experimental setup in the social condition and trial scheme. (**A**) In the social condition, participant and confederates were seated next to each other in front of the theatre screen. The recording device was located on a small box in front of them. In order to answer the questions after each scene, individuals had laptops on their lap. The laptops for stimulus presentation and for the physiological recording were placed in the back of the room. ECG was recorded from all individuals, GSR was recorded from the participant only due to limited input channels of the recording device. (**B**) Trial sequence for each film scene. After watching the scene, participants (and confederates in the social condition) answered several questions on VAS and in multiple-choice as well as dichotomous format. ECG = electrocardiogram; GSR = galvanic skin response, MC = multiple choice; VAS = visual analogue scale.

The experiment took place in the cinema “Z-inema, Berlin”. Participants were invited twice to take part in a ‘physiological experiment on film perception’, while they were sitting once alone in the cinema and once in a group. The order of sessions was counterbalanced across participants. The social condition consisted of three unfamiliar confederates, who were psychology students trained in pretending to be other participants taking part in the experiment. The gender ratio of the group was balanced on the basis of the participant’s gender. Altogether, there were four students (two female) acting as confederates so that the identities remained stable within the group over the course of the experiment. Upon arrival, a trained experimenter informed the participants about the scope of the study and the applied psychophysiological methods. Participants were told that the experimental procedure required that they turned off their phones and interacted as less as possible during the experiment in order to create a similar social situation in all sessions. Sitting in the lobby of the cinema, participants filled out the informed consent form (only first session), a questionnaire about demographic information (only first session) and the 20-items mood questionnaire PANAS (Cronbach’s α = .79) [49]. Afterwards, they were accompanied by the experimenter into the movie hall and connected to the physiological recording device, one by a time. In the social condition, the participant was seated in the middle, with a confederate of the same gender to the right, and two confederates of a different gender to the left (see Fig 1A). The experimenter explained to the participants that they would watch different short movie scenes on the movie screen and, subsequently, answer questions about them via a laptop. Afterwards, the experimenter showed the participants how to respond to upcoming questions with touchpad and key presses on the laptop placed on their lap. Moreover, he asked the participants to move as less as possible during the movie scenes, which was the window of interest for physiological data acquisition and analysis. Once the lights were turned off, the experimental protocol started with a two minutes baseline during which a fixation cross occurred on the movie screen. Thereafter ten movie scenes were shown in randomized order. After each scene, participants opened their laptops and responded with no time limit to (1) how much empathy they felt with the character on screen (e.g., “How strong was your feeling that Jenny was moved?”) via a continuous visual analogue scale (VAS) ranging from 0 to 100, (2) which emotion they felt themselves (multiple choice of ‘amusement’, ‘anger’, ‘fear’, ‘tenderness’, ‘neutral’) and (3) how strong they felt it (VAS), (4) how much they shared a true or false belief (correct or incorrect Theory of Mind) of the character on screen (e.g., correct: “How much do you think that the general wanted to hinder the crowd from hearing Forrest’s speech?”; incorrect: “How much do you think that the general wanted to give the speech himself?”) via VAS, (5) whether they remembered a (non-social) aspect of the scene (e.g., “Which word is written on the sign with the big red letters?”) via multiple choice, and (6) whether they had seen the movie before (yes/no). Note that for Theory of Mind, participants answered one correct and one incorrect question (order counterbalanced) for each scene, resulting in overall seven questions per scene. A typical trial scheme for one film scene is depicted in Fig 1B. The order of film scenes was randomized. The directionality of VAS scales was counterbalanced within participants. In the end of the experimental protocol, participants were detached from the physiological recording device and brought back to the lobby, where they filled out the Interpersonal Reactivity Index (IRI; [50]), a 28-items questionnaire (Cronbach’s α = .80) assessing empathy on four separate subscales: (1) perspective taking; (2) empathic concern; (3) personal distress; (4) fantasy. Before the payment of their compensation in the second session of the study, participants were debriefed that the other participants in the group condition were confederates. Questioning prior to debriefing revealed that all participants had been convinced that the confederates had been real participants. The film questionnaire on the laptop was programmed using the Psychophysics Toolbox extensions [51] for Matlab. The presentation of film scenes and their synchronization with physiological recording was programmed in E-prime 2.0 software (Psychology Software Tools, Inc). Unless noted otherwise, all inferential statistical analyses were performed in SPSS 24.0 (IBM Corp.) with Bonferroni correction for multiple comparisons.

### Stimuli

Ten film clips of two minutes length were selected to evoke amusement, anger, fear, tenderness or a neutral state. We chose all scenes based on emotion ratings of previous studies[19, 52–55] out of mainstream fictional Anglo-American productions. Only scenes from commercially available feature films displaying social situations were used in order to keep the scenes comparable regarding format and content. This also allowed for empathy and ToM questions even for the neutral film clips. All scenes were shown on the cinema screen (3,2 m diagonal) in standard 720p HDTV format. The sound was presented through the cinema sound system (5.1 with dolby surround system and low frequency enhancement). For each of the four emotions and for the neutral condition, two film scenes were selected which, in prior work, reliably elicited the respective emotion and which were similar regarding the film genre as well as the number of characters appearing in the scene. More details on selected film scenes and their use in previous studies can be found in S1 Table. All film clips used in the present study are available for research purposes upon request.

### Physiological recordings and preprocessing

Electrocardiogram (ECG) was measured by means of three Ag/AgCl pre-gelled electrodes with a contact area of 10 mm diameter that were placed in Einthoven’s triangle configuration (Powerlab and OctalBioAmp8/30, ADInstruments, UK). The signal was sampled with 2 kHz and online filtered via Mains Filter. After data acquisition, R-peaks were detected and R-R intervals extracted. Artifacts were rejected by detecting outlier points, typically caused by failure to detect an R-peak (‘edit via division’) or faulty detections of two or more ‘peaks’ within a period representing the R-R interval (‘edit via summation’). RSA amplitude was quantified as the variance of heart rate activity across the band of .12 - .4 Hz frequencies associated with spontaneous respiration [26] with CMetX[56]. RSA was assessed for the first 2 min baseline of each session and each 2 min film scene according to guidelines [57]. The RSA for each emotion condition was averaged across two film scenes. RSA response values for each emotion were baseline-corrected on the mean of the two RSA baseline values (i.e., social baseline and alone baseline). The same procedure was followed for both conditions (i.e., alone and social). Participants’ heart rate (HR) was computed as additional cardiac parameter found to be sensitive to the emotional valence of movies [14]. R-R intervals were converted to HR (beats per minute) using CMetX.

GSR was recorded at 2 kHz using Powerlab (ADInstruments, UK) and Ag/AgCl electrodes attached to the proximal phalanx of the left index and ring finger. Data were prepared and analysed using PsPM 3.1.1[44], a Matlab toolbox for model-based analyses of psychophysiological measures, available at pspm.sourceforge.net. Data preparation included scaling the data from S to μS, importing the files into PsPM, and trimming the data from 10 s before the first film scene to 60 s after the last one. Epoch files were created to indicate the respective time windows of the ten 2 min film sequences. As recommended for non-event-related designs[58], tonic sympathetic arousal during the epochs was estimated on the first level. Thus, first level models for tonic skin conductance level (SCL), i.e., the mean signal over the epoch, were calculated for each participant and each session. Default filter settings, containing a unidirectional Butterworth bandpass filter (cut of frequencies of .0159 Hz and 5 Hz) and down-sampling to 10 Hz, were applied[59].

## Results

### Cardiac results

**Fig 2:**
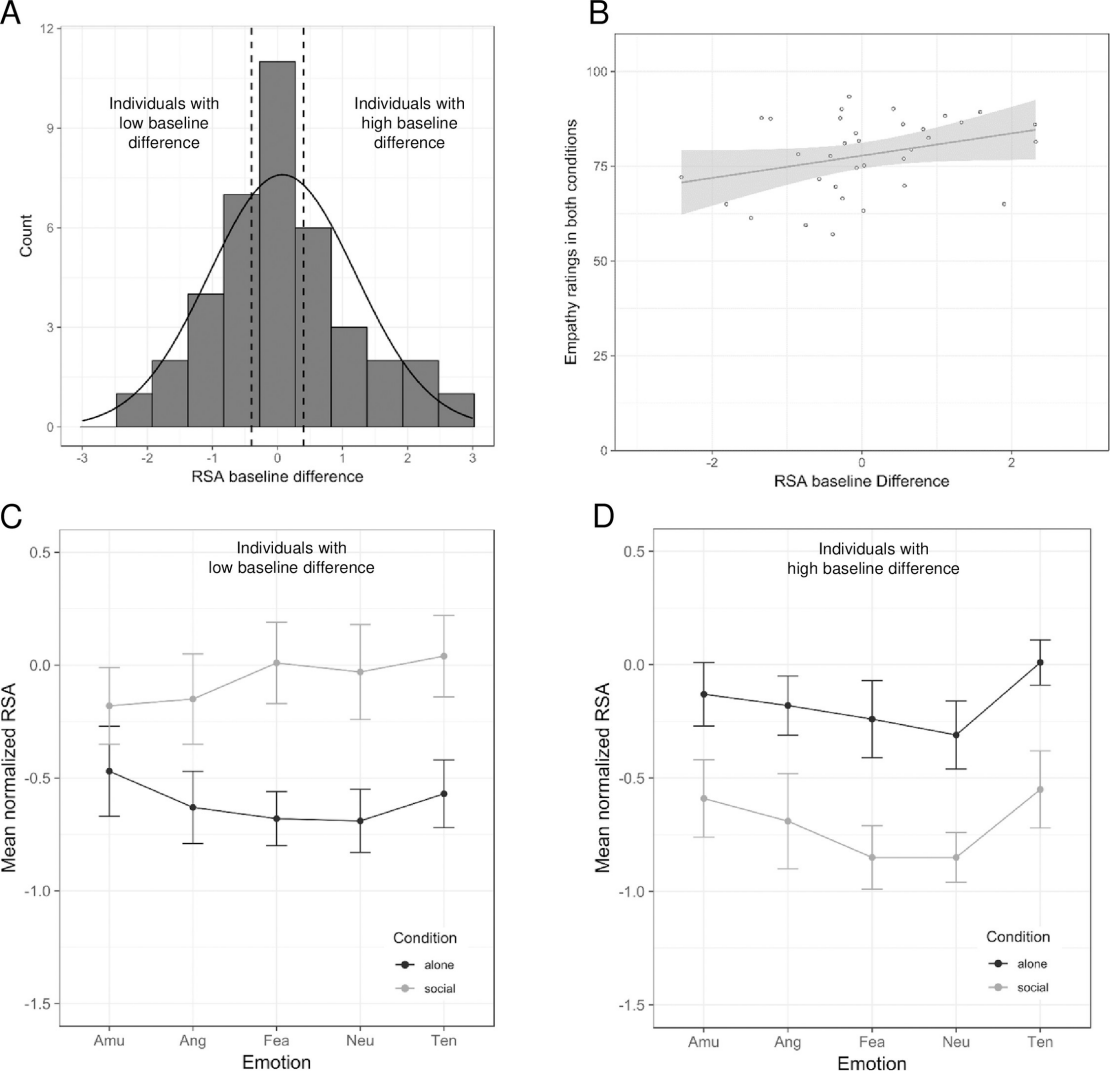
Results of the RSA analyses. (**A**) Distribution of baseline differences in RSA between social and alone condition. (**B**) Correlation between baseline differences in RSA and empathy ratings for characters in the film scenes (*r* = .32, *p* = .06). The shaded area represents the 95% confidence region. (**C, D**) Mean RSA values per emotion in both conditions in individuals with low RSA baseline differences (**C**) and in individuals with high RSA baseline differences (**D**), respectively. Amu = amusement; Ang = anger; Fea = fear; Ten = tenderness; Neu = neutral; RSA = respiratory sinus arrhythmia. Error bars represent the standard error of the mean.

The mean baseline RSA [expressed in ln(msec)^2^] prior to film screening did not significantly differ between alone [6.30,.18 (*M*, *SE*)], and social (6.23, .17) condition across all participants (*t*(38) = .418, *p* = .678). The absence of a significant difference in the mean baseline RSA between the two conditions is explained by the expected individual differences in tonic RSA flexibility. As clearly shown in the histogram (Fig 2A) participants’ ‘RSA baseline difference’ values–reflecting RSA baseline in the alone minus in the social condition–were normally distributed as shown by Kolmogorov-Smirnov test (p = .09). Accordingly, we split our total sample (*N* = 39) in three equally-dense subgroups of 13 participants each, based on data distribution: *individuals with low baseline difference* (i.e., social baseline RSA > alone baseline RSA, -1.04, .16); *individuals with high baseline difference* (i.e., alone baseline RSA > social baseline RSA, 1.3, .16) and *individuals with no baseline difference* (-.05, .16). To ensure that RSA baseline differences driving the subgroups splitting were not due to a mere condition order effect, Chi-square test was performed on the number of participants who did first the alone and then social condition as a function of subgroup. The difference was not significant, (χ^2^(2, *N* = 39) = 3.28, *p* = .19). In order to address our main hypothesis about the role of vagal flexibility in response to others’ mere presence during the enjoyment of film clips, we included the between-subject factor ‘baseline RSA’ (*high baseline differences vs*. *low baseline differences vs*. *no baseline differences–*see above) into the subsequent ANOVA on RSA during film viewing with the within-subject factors ‘condition’ (alone vs. social) and ‘emotion’ (amusement vs. anger vs. fear vs. tenderness vs. neutral). Results showed a significant main effect of ‘emotion’ (*F*(4,144) = 3.2, *p* = .015, *ηp*^*2*^ = .08) and an interaction effect ‘condition*baseline RSA’ (*F*(2,36) = 14.37, *p* < .001, *ηp*^*2*^ = .44). For the main effect of ‘emotion’, Bonferroni-adjusted pairwise comparisons yielded non-significant differences (0.08 < *p* < 1.0). Mean RSA values for all emotions and conditions are shown in Table 1. Specifically, Bonferroni corrected post-hoc comparisons revealed that for participants with high baseline differences (alone baseline RSA > social baseline RSA), RSA responses during film viewing were significantly higher (*p* < .001) in the alone condition (-.17, .12) than in the social condition (-.71, .15). Conversely, participants with low baseline difference (social baseline RSA > alone baseline RSA) had significantly higher RSA values (*p* < .001) while watching the scenes in the social (-.06, .15) compared to the alone condition (-.61, .12) (Fig 2C and 2D). No significant difference was found for no baseline difference group (*p* = .81).

**Table 1:** Descriptive statistics for RSA data per emotion in both conditions.

Emotion	Condition	RSA	
		*M*	*SE*
Amusement	*alone*	-.30	.1
	*social*	-.36	.09
Anger	*alone*	-.41	.08
	*social*	-.41	.11
Fear	*alone*	-.44	.08
	*social*	-.40	.09
Tenderness	*alone*	-.29	.09
	*social*	-.25	.09
Neutral	*alone*	-.45	.08
	*social*	-.40	.09

*M* = mean; *RSA* = respiratory sinus arrhythmia; *SE* = standard error.

In the repeated-measures ANOVA on HR we included as between-subject factor ‘baseline RSA’ (high baseline differences vs. low baseline differences vs. no baseline differences) and as within-subject factors ‘condition’ and ‘emotion’. Results showed a significant main effect of ‘emotion’ (*F*(4,144) = 28.46, *p* < .001, *ηp*^*2*^ = .44). Bonferroni comparisons yielded significant higher HR for tender scenes than all the other ones (Amusement: -4.88, .65; Anger: -4, .68; Fear: -3.06, .55; Tenderness: -1.49, .54; Neutral: -4.81, .60; all *Ps* < .001). Fearful clips elicited higher HR with respect to amusing and neutral movies (*Ps* = .001).

### GSR results

**Fig 3:**
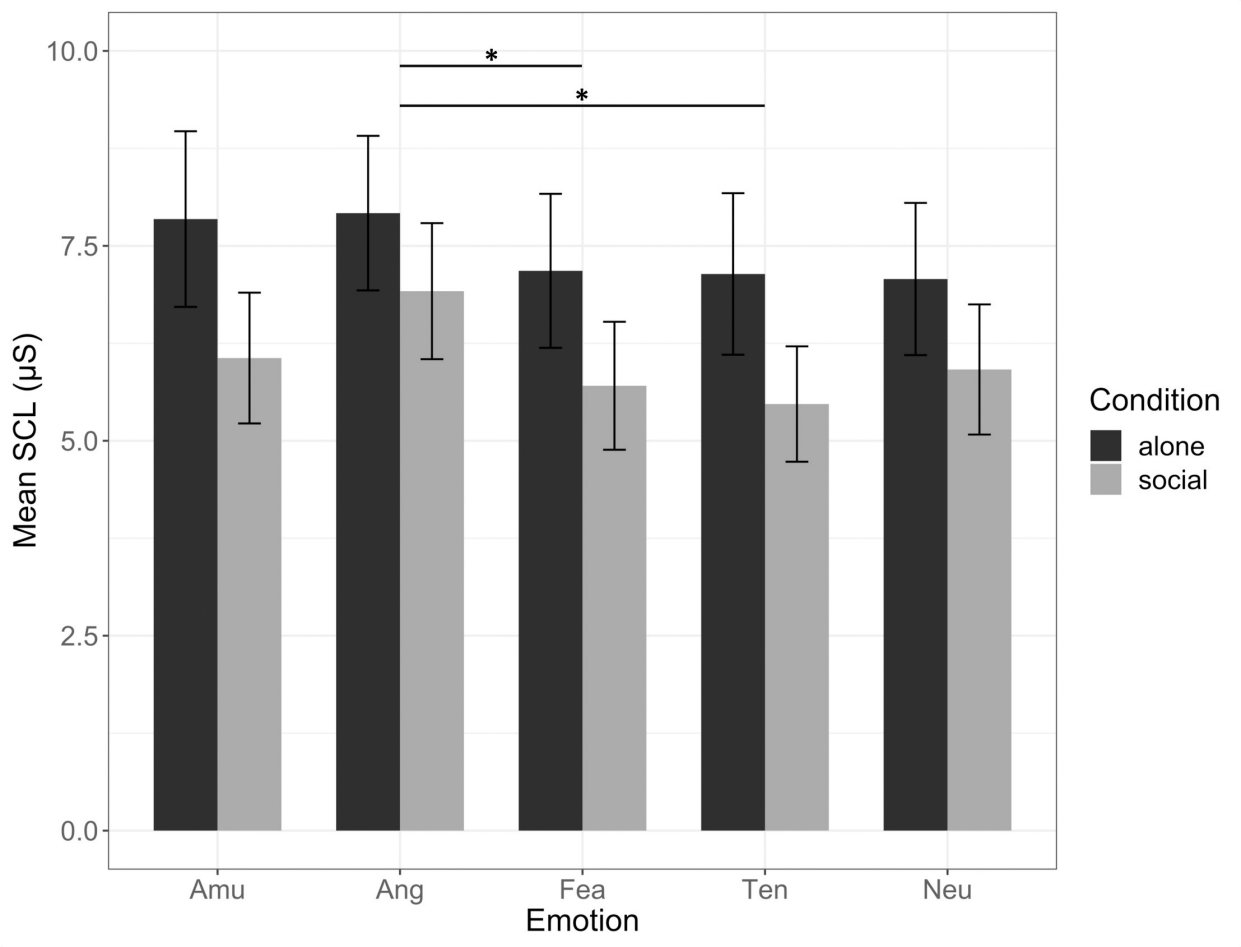
SCL values per emotion in both conditions. Error bars represent the standard error of the mean. Amu = amusement; Ang = anger; Fea = fear; Ten = tenderness; Neu = neutral; SCL = skin conductance level.

To compare physiological arousal as reflected in the SCL parameter of GSR (see Fig 3), repeated-measures ANOVA was conducted including the between-subject factor ‘baseline RSA’ (high baseline differences vs. low baseline differences vs. no baseline differences–see above) and the within-subject factors ‘condition’ and ‘emotion’. Results showed a significant main effect of ‘emotion’ (*F*(3.2,115) = 3.89, *p* = .01, *ηp*^*2*^ = .097). Bonferroni comparisons showed significantly higher SCL values during angry scenes (7.42, .74) than during scenes evoking fear (6.44, .7) and tenderness (6.3, .77).

### Behavioural results

Behavioural data of one participant in the alone session and of five participants in the social condition were lost due to software malfunction. For the remaining *behavioural* data (*N* = 33), five separate repeated-measures ANOVAs were conducted including the between-subject factor ‘baseline RSA’ (high baseline differences vs. low baseline differences vs. no baseline differences–see above) and the within-subject factors ‘condition’ (alone vs. social) and ‘emotion’ (amusement vs. anger vs. fear vs. tenderness vs. neutral). For each ANOVA, dependent variables (DVs) corresponded to the participants’ explicit ratings on their laptops regarding empathy, emotion intensity, ToM (correct or incorrect) and memory.

Descriptive statistics of the ratings in both conditions over all emotions are depicted in Table 2. Considering empathy ratings, repeated-measures ANOVA showed a significant main effect of the factor group (F(2,30) = 3.63, *p* = .04, *ηp*^*2*^ = .19) and emotion (F(2.98,89.41) = 10.72, *p* < .001, *ηp*^*2*^ = .26). Bonferroni comparisons conducted on the main effect of group demonstrated near to significant higher empathy ratings for high baseline differences group than low baseline differences group (high baseline differences group = 81.36, 2.74; low baseline differences group = 71.6, 2.86; *p* = .058). Bonferroni post-hoc on the main effect of emotion revealed that participants gave the highest empathy ratings to fearful scenes significantly more than for amusing, neutral and tender ones (all *Ps* < 0.04). Lastly, empathy ratings given to amusing movies were significantly lower than those given to tender scenes (*p* = 0.03). Regarding emotion intensity ratings, repeated-measures ANOVA showed a significant main effect of the factor emotion (F(3,90.03) = 27.66, *p* < .001, *ηp*^*2*^ = .48). Bonferroni post-hoc on the main effect of emotion revealed that participants gave the lowest emotion intensity ratings to neutral scenes (all *Ps* < .001). Considering correct TOM ratings, repeated-measures ANOVA showed a significant main effect of the factor emotion (F(2.44,73.12) = 24.15, *p* < .001, *ηp*^*2*^ = .45). Bonferroni post-hoc on the main effect of emotion showed that participants gave different correct TOM to all scenes, with the exception of amusement vs. fear (*p* > .05) and tenderness vs. neutral (*p* > .05). Differently, Bonferroni comparisons conducted on the significant main effect of the factor emotion (F(4,8) = 29.03, *p* < .001, *ηp*^*2*^ = .49) for incorrect TOM ratings showed significant higher scores for tenderness than all the other emotions (all *Ps* < .001). No significant results were found for memory dependent variable.

**Table 2:** Descriptive statistics for the behavioural data in both conditions.

	*Emotion*	*M*	*SE*	*CI 95%*
**Empathy**	Amu	67.96	3.51	60.78–75.14
	Ang	79.89	2.23	75.32–84.44
	Fea	87.72	2.41	82.78–92.65
	Ten	80.90	2.64	75.51–86.3
	Neu	72.72	2.34	67.95–77.5
**Emotion Intensity**	Amu	55.06	3.93	47.03–63.1
	Ang	64.46	3.1	58.13–70.8
	Fea	53.05	4.54	43.8–62.31
	Ten	64.42	3.74	56.78–72.07
	Neu	24.13	4.02	15.92–32.34
**ToM *(correct)***	Amu	80.75	2.47	75.7–85.8
	Ang	64.73	3.6	57.4–72.06
	Fea	79.3	2.99	73.18–85.41
	Ten	90.53	1.43	87.61–93.45
	Neu	91.22	1.51	88.14–94.3
**ToM *(incorrect)***	Amu	30.52	3.11	24.17–36.88
	Ang	21.88	2.84	16.07–27.7
	Fea	28.4	2.82	22.64–34.17
	Ten	58.95	3.64	51.51–66.38
	Neu	28.41	3.11	22.05–34.77
**Memory**	Amu	0.89	0.02	0.84–0.94
	Ang	0.75	0.05	0.65–0.85
	Fea	0.82	0.04	0.74–0.89
	Ten	0.78	0.04	0.69–0.86
	Neu	0.79	0.04	0.71–0.89

*M* = mean; *SE* = standard error; CI 95% = Confidence Interval 95%; Amu = Amusement; Ang = Anger; Fea = Fear; Ten = Tenderness; Neu = Neutral.

## Discussion

The aim of the present study was twofold: to understand how social company affects empathetic processes elicited by film and to investigate the role of vagal flexibility to others’ mere presence during the enjoyment of movie clips. To this purpose, we acquired verbal reports about the emotions elicited in the participants as well as implicit psychophysiological measures (RSA, HR, GSR) of socio-emotional responses, contrasting solitary and social film reception in a small cinema.

Considering our main cardiac measure, respiratory sinus arrhythmia (RSA), we found an interesting mediating effect of vagal flexibility to the mere presence of others. Individuals that showed a high baseline difference (alone vs. social) prior to the presentation of the film clips, expressed higher RSA in the alone compared to the social condition. Conversely, low baseline difference participants showed lower RSA for the alone condition while watching the clips. The social condition therefore had differential effects for those two groups. These results confirm our hypothesis that people diverge in the way they modulate their vagal responses to the mere presence of others. Interestingly, such modulation in response to social cues influences also how people engage in more complex contexts such as the enjoyment of movie clips. According to polyvagal theory [28, 29, 38] higher amplitude of tonic RSA reflect self-regulatory parasympathetic relaxation, thereby facilitating engagement with (social) stimuli in the environment, which can also be conceptualized as a trait [60]. In this respect, the results of the present study suggest that besides the amplitude of tonic RSA its flexibility to the mere presence of others is particularly informative about the subsequent vagal regulation in social contexts. As stated in the introduction, people join cinematic experiences with the intention to collectively attend to the same object. Proceeding from the present results, we can only hypothesize that people who increase their vagal modulation to the mere presence of others (i.e., low baseline difference group) will enjoy more collective film reception in a movie theatre than people who do not (i.e., high baseline difference group). Actually, the potential substrates of collective aesthetic experiences are an under investigated field of research which can be enriched by ecologically valid experimental physiological approaches as ours.

With our study being the first attempt to measure tonic RSA flexibility to the mere presence of others, further attempts are required to confirm and extend the validity of the tonic RSA flexibility as a predictor of spontaneous vagal regulation in more interactive social contexts. Moreover, it will be interesting to investigate the potential relation between tonic RSA flexibility and individual psychological traits.

Contrary to our expectation, our RSA results did not show either a specific modulation in function of the emotional content of the movie clips or an interaction with participants’ group. Previous studies showed both specificity and similarity of autonomic activity in emotions [61]. The negative result found here does not speak against a physiologically differential response to different emotions. Indeed, HR and GSR results showed some attended modulation in response to movies emotional content [46, 47, 62], in negative film genre such as horror eliciting stronger physiological arousal. The absence of a modulation of HR and GSR in function of the here demonstrated vagal flexibility in response to social contexts suggests that art enjoyment in solitary or social conditions specifically influences the individual vagal regulation, associated with social disposition, and not people’s broader autonomic reactions. From this point of view, future work on the film social audience effect [6] could extend the distancing-embracement model of the enjoyment of negative emotions which has been proposed across art genres [3]. The model proposes processing mechanisms of psychological distancing implying personal safety and control during exposure to art and fiction involving negative emotions. The distancing mechanism, which could involve differential responses of the ANS, allows the recipient to positively embrace the experience of negative emotions, thereby rendering art reception more profound and emotionally moving. Importantly, the distancing-embracing mechanisms include compositional interplays of positive and negative emotions, of activation (sympathetic) and deactivation (parasympathetic) of the ANS. Recent evidence on the reception of poetry suggests that strict antagonistic responses of the sympathetic and parasympathetic systems according to valence or arousal fail to explain the psychophysiology of socio-emotional aesthetic processes [63].

In the explicit measures, contrary to our hypotheses, we did not find a main effect of group, nor an interaction of group with social condition for emotion intensity, TOM and memory. Differently, empathy ratings were higher for high baseline differences group than low baseline differences group. In other words, people who decrease their baseline RSA in response to others’ company are those who felt themselves more empathically engaged with the characters. Although the present study cannot provide a clear-cut interpretation of this result, we could hypothesize that high baseline differences people feel the presence of others as a potential social stress, which they tackle by developing stronger explicit empathetic access to others’ mental states, here reflected in the empathy ratings about the movie characters’ feelings. Furthermore, the expected modulation of social company on the explicit measures was not confirmed by the present results, suggesting that the presence of few unfamiliar people behaving in a neutral attitude does not influence the explicit evaluation of the movie scenes. Differently, we found evidence for emotion-specific effects on reported empathic responses to the protagonists, on emotion intensity and on Theory of Mind.

Reported empathy for the actors was found significantly higher for film clips of negative emotion as fear than for film clips of positive or neutral emotion. This is compelling with respect to psychological research proposing that negative emotions have a distinct potential for capturing attention [64], providing a high intensity of subjective feeling [65] and privileged storage in memory [66, 67]. Negative emotions seem to be not only a key factor for the evolution of neuronal circuits underlying survival mechanisms [68], but also for interpersonal [69] and prosocial behaviour [70, 71]. The assumption that negative emotions promote social cohesion has been the focus of anthropological work studying ritual practices of pain in different cultures around the world [72, 73]. Several independent lines of research suggest that negative emotional experiences can foster an increase of empathetic and affiliative behaviour [74, 75], particularly within the in-group [76]. Ratings of emotion intensity were found significantly lower for neutral scenes than all the other ones, irrespectively to the quality of their emotional content, suggesting that spectators’ engagement with positive and negative clips was comparable. Interestingly, perspective-taking in terms of true beliefs (‘correct ToM’) was more pronounced in film scenes displaying positive or neutral content compared to negative content. Spectators were more able to detect others’ correct intentions when acted in a positive or neutral context. Differently, wrong perspective-taking (‘incorrect ToM’) was more prominent in response to tender scenes, likely indicating that intentions behind a tender context were more difficult to be interpreted.

These results are in line with recent studies on the psychological construct of “being moved” [55, 77], which has been described as a mixed emotion involving joy and sadness associated with a low-to-mid level of physiological arousal and subjective experiences of high intensity. According to Menninghaus and his colleagues, a great amount of tenderness eliciting scenarios can be assigned to significant relationship and critical life events as birth, death, separations, reunifications, film and music, which is also reflected in our result of mean HR as an additional indicator of physiological arousal, being most pronounced during tender scenes. “In all these instances, one’s own agency and causation by one’s own behavior have relatively little importance for the elicitation of feelings of being moved; rather, an (empathic) observer or witness perspective prevails” [77].

We believe that these behavioral results importantly contribute to the ongoing debate about the function of emotions in socio-cultural practices, especially when integrated with the here found physiological reactions to social situations.

Although the present study used a controlled design with balanced and ecological valid conditions, it has some limitations that need to be acknowledged. First, it is noteworthy that our main manipulation, the social context, failed to reach significant effects in most of our measures. While there are methodological reasons that may partly explain the absence of effects in the measures (lack of power due to asymmetrical data loss in ratings, sociality of all depicted scenes), we believe that our results shed light on the differentiated nature of the social facilitation effect. It seems that sharing a socio-cultural practice with passive strangers, as in our experimental (confederate) set-up, was not powerful enough to modulate empathetic responses in explicit and implicit cardiac measures. We believe that sharing the filmic experience includes additional nonverbal ways of sharing emotions with our companions such as weeping together, laughing out loud, consoling somebody, touching each other and exchanging glances [78]–influences that we controlled in this first empirical investigation of the social audience effect by employing a set-up with three unfamiliar confederates. It is possible that we could have found stronger physiological and behavioural emotional responding, if we had used confederates familiar to the participant, since emotional contagion and empathy are dependent on familiarity and similarity with the other [79, 80]. Second, our experimental set-up did not allow for the assessment of the subjective experience and their interplay with physiological measures during the movie, as we only assessed ratings after each scene in order not to disturb the physiological assessment. Third, it might have been of interest to look into gender differences of socio-emotional responding [81], however the investigation of group differences in vagal regulation in terms of baseline differences prevented us from having an additional between-subject factor, due to power issues. Fourth, while we did not assess movie preferences, we tested whether participants had seen the movie previously, which did not have a significant effect onto any of our DVs. Future research should systematically vary nonverbal exchanges and the dynamics[82] of the subjective experience during social film reception.

To conclude, our data reveal a specific role of the vagal regulation in response to the mere presence of others in explicit empathic engagement with characters and in parasympathetic response to shared filmic experience. Researching the sharing of emotional experiences in film requires the analysis of a complex system of dynamic relations between aspects of films and their spectators[83].

## Supporting information

S1 Table Film scene selection for each emotion including emotion ratings of previous studies. *Note*: *min* = lowest value; *max* = highest value.
^a^ Cuts with respect to the film clips used in the previous studies. Negative signs (*-*) indicate the time before the original sequence started, positive signs (*+*) the time after the original sequence ended.
^b^ Film clip was not provided by the previous study. Therefore, exact timings of the new cuts are not available. Cuts were performed based on content description and coherence. All film clips used in the present study are available for research purposes upon request.
(DOCX)
