# Supplementary material for: Sharing the filmic experience - The physiology of socio-emotional processes in the cinema
Source: PLoS One. 2019 Oct 18;14(10):e0223259. doi: 10.1371/journal.pone.0223259 (PMC6799930; doi:10.1371/journal.pone.0223259)
Supplement: S1 Table — Note: min = lowest value; max = highest value. a Cuts with respect to the film clips used in the previous studies. Negative signs (-) indicate the time before the original sequence started, positive signs (+) the time after the original sequence ended. b Film clip was not provided by the previous study. Therefore, exact timings of the new cuts are not available. Cuts were performed based on content description and coherence. All film clips used in the present study are available for research purposes upon request. (DOCX) [file pone.0223259.s001.docx]

# **Supporting Information**

for

**Sharing the filmic experience - the physiology of socio-emotional processes in the cinema**

Laura Kaltwasser, Nicolas Rost, Martina Ardizzi, Marta Calbi, Luca Settembrino, Joerg Fingerhut, Michael Pauen, Vittorio Gallese

**Stimuli**

The film clips for eliciting amusement were scenes from *A Fish Called Wanda* (1988; Charles Crichton) and *There’s Something About Mary* (1998; Bobby Farrelly). The scenes for tenderness were both taken from *Forrest Gump* (1994; Robert Zemeckis), the scenes for anger from *Witness* (1985; Peter Weir) and *Crash* (2004; Paul Haggis), the scenes for fear from *The Shining* (1980; Stanley Kubrick) and *Scream* (1996; Wes Craven), and the neutral scenes from *All the President’s Men* (1976; Alan J. Pakula) and *Omagh* (2004; Pete Travis). The film clip selection including a short description and their emotion ratings in previous studies are listed in Table S1. Small adjustments of duration were necessary for some scenes in order to keep the consistent length of 2 min and to create a coherent and sensible content.

**Table S1**. Film scene selection for each emotion including emotion ratings of previous studies. *Note: min* = lowest value; *max* = highest value.

| Emotion | Film title | Scene content | Previous study | Emotion rating (*min-max*) | Duration in previous study | New cut**^a^** |
| --- | --- | --- | --- | --- | --- | --- |
| Amusement | A Fish Called Wanda | *Archie is found naked by the owners of the house.*  *First scene: Archie recites Russian poetry.*  *Last scene: Archie says to the family: “How nice to see you.”* | Schaefer et al. (2010)[^1^](#_ENREF_1) | 5.11 (1-7) | 3:01 min | 0:58 – +0:01 |
|  | There’s Something About Mary | *Ted fights with a dog.*  *First scene: Something is thrown into Magda’s drink.*  *Last scene: Magda and Mary leave the flat screaming. Ted lies on the floor.* | Schaefer et al. (2010)[^1^](#_ENREF_1) | 4.85 (1-7) | 2:55 min | -0:09 – 0:01,  0:27 – 1:15,  1:37 – 2:10,  2:39 – 2:55^b^ |
| Anger | Crash | *A cop pulls over a black couple and sexually assaults the wife in front of her husband.*  *First scene: Cameron says to Christine: “I’m o.k., I got this.”*  *Last scene: Cameron says to the police officers: “We would appreciate it if you’d just let us go with a warning.”* | Bartolini (2011)[^2^](#_ENREF_2) | 6.9 (0-8) | 6:33 min | N/A^b^ |
|  | Witness | *A group of Amish is harassed by teenagers.*  *First scene: Eli and John ride a horse-drawn carriage.*  *Last scene: John puts his hat back on his head.* | Hewig et al. (2005)[^3^](#_ENREF_3) | 5.34 (0-9) | 1:31 min | N/A^b^ |
| Fear | Scream | *A girl receives threats over the phone and is pursued by a murderer.*  *First scene: Casey speaks to the murderer on the phone, she says: “Please leave me alone.”*  *Last scene: Casey punches the murderer through a broken window.* | Schaefer et al. (2010)[^1^](#_ENREF_1) | 4.45 (1-7) | 6:36 min | 4:07 – 6:07 |
|  | The Shining | *A man pursues his wife and son with an axe.*  *First scene: Jack reaches through a broken door and unlocks it.*  *Last scene: Wendy hides in her bathroom holding a knife and screams while an axe breaks through the door.* | Schaefer et al. (2010)[^1^](#_ENREF_1) | 4.54 (1-7) | 6:15 min | 1:52 – 3:53 |
| Tenderness | Forrest Gump (1) | *Father and son are reunited.*  *First scene: Jenny is holding her son and closes the door after saying goodbye to a woman.*  *Last scene: Jenny breathes heavily while looking at Forrest and her son watching TV.* | Schaefer et al. (2010)[^1^](#_ENREF_1) | 5.96 (1-7) | 2:01 min | -0:05 – 1:55 |
|  | Forrest Gump (2) | *Forrest is reunited with Jenny at the Lincoln Memorial.*  *First scene: The crowd cheers as Forrest is standing behind many microphones. After a few seconds, he starts with: “Well…”*  *Last scene: Forrest and Jenny hug in the reflecting pool, the crowd cheers.* | Wassiliwizky et al. (2015)[^4^](#_ENREF_4) | 3.13 (0-4) | 0:58 min | N/A^b^ |
| Neutral | All the President’s Men | *During a hearing, a reporter asks one of the attendees what had just happened.*  *First scene: Markham is sitting in a courtroom, Mr. Woodward sits down in the row behind him.*  *Last scene: Markham and Mr. Woodward are talking in a hallway, Markham leaves with the words: “I just don’t have anything to say.”* | Hewig et al. (2005)[^3^](#_ENREF_3) | All emotion ratings < 2.50 (0-9) | 1:05 min | N/A^b^ |
|  | Omagh | *A father and son are fixing a car, the son leaves to go shopping with a friend.*  *First scene: Aiden lies under a car, this father puts aside his mug and joins him.*  *Last scene: Aiden and Michael are driving in a car. The town sign of Omagh appears on the left side.* | Jenkins & Andrewes (2012)[^5^](#_ENREF_5) | All emotion ratings < 1.2 (0-9) | 1:00 min | N/A^b^ |

^a^ Cuts with respect to the film clips used in the previous studies. Negative signs (*-*) indicate the time before the original sequence started, positive signs (*+*) the time after the original sequence ended.

^b^ Film clip was not provided by the previous study. Therefore, exact timings of the new cuts are not available. Cuts were performed based on content description and coherence. All film clips used in the present study are available for research purposes upon request.

**References**

1 Schaefer, A., Nils, F., Sanchez, X. & Philippot, P. Assessing the effectiveness of a large database of emotion-eliciting films: A new tool for emotion researchers. *Cognition & Emotion* **24**, 1153-1172, doi:10.1080/02699930903274322 (2010).

2 Bartolini, E. E. *Eliciting Emotion with Film: Development of a Stimulus Set* Bachelor of Arts thesis, Wesleyan University, (2011).

3 Hewig, J. *et al.* A revised film set for the induction of basic emotions. *Cognition & Emotion* **19**, 1095-1109, doi:10.1080/02699930541000084 (2005).

4 Wassiliwizky, E., Wagner, V., Jacobsen, T. & Menninghaus, W. Art-elicited chills indicate states of being moved. *Psychology of Aesthetics, Creativity, and the Arts* **9**, 405-416, doi:10.1037/aca0000023 (2015).

5 Jenkins, L. M. & Andrewes, D. G. A New Set of Standardised Verbal and Non-verbal Contemporary Film Stimuli for the Elicitation of Emotions. *Brain Impairment* **13**, 212-227, doi:10.1017/BrImp.2012.18 (2012).
